# Effects of water stress on spectral reflectance of bermudagrass

**DOI:** 10.1038/s41598-020-72006-6

**Published:** 2020-09-14

**Authors:** Lisa Caturegli, Stefania Matteoli, Monica Gaetani, Nicola Grossi, Simone Magni, Alberto Minelli, Giovanni Corsini, Damiano Remorini, Marco Volterrani

**Affiliations:** 1 Department of Agriculture, Food and Environment, University of Pisa, Pisa, Italy; 2 Institute of Electronics, Computer and Telecommunication Engineering, National Research Council of Italy, Pisa, Italy; 3 Department of Agricultural and Food Sciences, University of Bologna, Bologna, Italy; 4 Department of Information Engineering, University of Pisa, Pisa, Italy

**Keywords:** Imaging and sensing, Agroecology, Plant ecology, Plant sciences

## Abstract

In the south-central Italy, during summer rainfall does not supply a sufficient amount of water. Therefore, irrigation management during dry periods is important for maintaining turf quality. The hybrid bermudagrass (*Cynodon dactylon* (L.) Pers. × *Cynodon transvaalensis* Burtt–Davy) is known to represent the dominant warm-season turfgrass in warm to temperate climatic regions and its drought tolerance make bermudagrass a competitive turfgrass. A greenhouse experiment was conducted using uniform cores of hybrid bermudagrass, which were secured in a polyvinyl chloride cylinders and watered by constant sub-irrigation. The objectives of the present research were to measure the spectral reflectance with a new generation handheld spectroradiometer on hybrid bermudagrass and to explore various vegetation indices to be used as future detecting tool to study water stress in bermudagrass. Moreover, the potential uses of multivariate processing techniques for discriminating different water stress conditions in turfgrass has been investigated. Besides spectral indices, multivariate methods, although performed on a data set limited in terms of sample size, have shown a great potential for water stress monitoring in turfgrass and surely deserve further investigations. There are different indices that use distinct water absorption features independent of chlorophyll concentration, such as water index (WI = R900/R970) that has been reported to be a robust index of canopy water content and is used as an active indicator of changes in Leaf Relative Water Content (LRWC). Also, the ratio of WI with NDVI (WI/NDVI = (R_900_/R_970_)/((R_800_ − R_680_)/(R_800_ + R_680_)]) was found to be an effective indicator of water stress. Another vegetation index to detect water features is normalized difference water index (NDWI), designed to maximize reflectance of water by using green wavelengths. In our trial in bermudagrass the relationships studied, suggest that WI (900/970) and WI/NDVI, among the indices studied, are the more effective indicators of water stress. In fact, lower values of WI indicate higher water stress, while higher values of WI/NDVI indicate higher water stress levels.

## Introduction

Climate change and the sustainability on the use of resources is at the heart of international topics and discussions. The trend is heading towards an optimization of inputs, such as irrigation and fertilization, also in the management and maintenance of turfgrasses.

In Italy, especially in the south-central areas, during summer rainfall does not supply a sufficient amount of water. Therefore, irrigation management during dry periods is important for maintaining turf quality^1^, in addition to an optimal turfgrass mowing management^2^. Thus, water conservation and availability are important and critical priorities for the turfgrass management^3^.

In the last two decades, several research works conducted in Southern Europe have investigated the adaptability of warm-season turfgrass species, including bermudagrass, to the Mediterranean environment^4–8^. Bermudagrass is known to represent the dominant warm-season turfgrass in warm to temperate climatic regions of the world and its drought tolerance makes bermudagrass an ideal and competitive turfgrass in many environments^9–13^.

In the past years several studies have been carried out to provide different methods for estimating the vegetation water content at different scales: ground-based or remotely sensed measurements^14,15^. For example, the analysis of the radiation reflected from the canopies can provide information about the water status of the vegetation that generated it and can be used in remote sensing to determine a possible water deficiency^16^. Thus, precision turfgrass water management, which combined the use of time-domain reflectometry (TDR) and spectral reflectance mapping has been proposed as an alternative method to improve irrigation efficiency^3,17^.

Several indices proposed in literature can quantify chlorophyll concentration^18^ and then allow remote detection methods to identify and map vegetation stress through the influence of chlorophyll content variation. In these studies and other articles^1,17,19,20^ the normalized difference vegetation index (NDVI)

$${\text{NDVI}} = \left( {{\text{R}}_{{{\text{NIR}}}} - {\text{R}}_{{{\text{red}}}} } \right)/\left( {{\text{R}}_{{{\text{NIR}}}} + {\text{R}}_{{{\text{red}}}} } \right)$$

(where R_NIR_ = reflectance in the near infrared region, and R_red_ = reflectance in the red region), was considered as the most commonly used reflectance index of relative plant health and stress indicator^19,21–25^. Thus, Jiang et al.^26^ and Johnsen et al.^22^ studied the relationship between NDVI and soil moisture. Although NDVI is able to evaluate water stress and uses narrow bands that overlap with chlorophyll features^27^, there are several indices that use distinct water absorption features regardless of the chlorophyll concentration. In fact, these indices are based on spectra bands located in the near-infrared region (NIR 750–1,300 nm) and short-wavelength infrared (SWIR 1,300–2,500 nm)^14,16,28–31^ with centers around 970, 1,240, 1,450, 1,950, 2,130 nm. Water index (WI)

$${\text{WI}} = {\text{R}}_{{{9}00}} /{\text{R}}_{{{97}0}}$$

has been reported to be a robust index of canopy water content^28,29^ and is used as an active indicator of changes in Leaf Relative Water Content (LRWC), especially when there are no important differences in canopy architecture^14^. To improve WI as LRWC indicator^32^ successfully tried to test the ratio of WI with NDVI

$${\text{WI}}/{\text{NDVI}} = \left( {{\text{R}}_{{{9}00}} /{\text{R}}_{{{97}0}} } \right)/\left( {\left( {{\text{R}}_{{{8}00}} - {\text{R}}_{{{68}0}} } \right)/\left( {{\text{R}}_{{{8}00}} + {\text{R}}_{{{68}0}} } \right)} \right)$$

with the aim of minimizing structural effects and then maximizing sensitivity to water content. Another vegetation index to detect water features is normalized difference water index (NDWI), designed to maximize reflectance of water by using green wavelengths, minimize the low reflectance of NIR by water features, and take advantage of the high reflectance of NIR by vegetation and soil features^30,33–36^. NDWI proposed by Gao^30^ considered two NIR bands, one centered at around 860 nm and the other at 1,240 nm

$${\text{NDWI}}_{{{124}0}} = \left( {{\text{R}}_{{{86}0}} {-}{\text{R}}_{{{124}0}} } \right)/\left( {{\text{R}}_{{{86}0}} + {\text{R}}_{{{124}0}} } \right)$$

and is mostly used for remote sensing of vegetation liquid water from space^37^. Moreover, several studies by Wu et al.^38^ and Zhang et al.^39^ have demonstrated the ability of this index to assess vegetation water content, not only from space as proposed by Gao^30^, but also from ground measurements. Also, Chen et al.^31^ studied NDWI to estimate vegetation water content in different crops, selecting bands centered in the NIR at around 860 nm and in the SWIR at 2,130 nm

$$({\text{NDWI}}_{{{213}0}} = \left( {{\text{R}}_{{{86}0}} {-}{\text{R}}_{{{213}0}} } \right)/\left( {{\text{R}}_{{{86}0}} + {\text{R}}_{{{213}0}} } \right).$$

Besides spectral indices, multivariate methods have also been explored to monitor various kind of stress in vegetation^40–42^. Among the different methods, Principal Component Analysis (PCA)^43^ has been employed as an unsupervised analysis of the data^40,42^, Linear Discriminant Analysis (LDA)^43^ has been employed as a supervised classification method^42^, and Principal Component Regression (PCR) and Partial Least Square (PLS) regression^44^ have been employed for multivariate regression in retrieval applications^45^. Supervised techniques such as LDA and PLS require a set of “training data” characterized by known stress conditions and are generally applicable when a statistically significant high number of spectral measurements is available.

The objectives of this work were (i) to measure the spectral reflectance with a new generation handheld spectroradiometer on a turfgrass species; (ii) to explore various narrowband vegetation indices that could be useful to detect water stress in bermudagrass; (iii) to study the relationship between selected indices and turfgrass water stress to be used as a future detecting tool, (iv) to investigate the potential of using multivariate processing techniques for discriminating different water stress conditions in turfgrass.

## Materials and methods

A greenhouse experiment was conducted at the Department of Agriculture, Food, and Environment of Pisa University, Pisa, Italy (lat. 43_40′N, long. 10_19′E, 6 m elevation), in May 2018, using uniform cores (9.6 cm diam. by 15 cm deep) of hybrid bermudagrass (Cynodon dactylon (L.) Pers. × Cynodon transvaalensis Burtt–Davy ‘Patriot’) collected on May 15, 2018 with undisturbed soil profile. In the glass greenhouse (the size of 16 × 6 m, oriented east west) the temperature, measured with a maximum–minimum thermometer, ranged from 34 to 19 °C (respectively day and night) with daily maximum photosynthetically active radiation (PAR) levels (measured with a LICOR 190R Quantum Sensor) ranging from 950 to 1,425 µmol m^−2^ s^−1^, provided by sunlight. For the research, the turfgrass cores were collected from a hybrid bermudagrass mature stand (> 5 years) that was selected at the Center for Research on Turfgrass for Environment and Sports (CeRTES) in S. Piero a Grado, (43°40′ N, 10°19′ E, 6 m. a.s.l.), at the Department of Agriculture, Food, and Environment of Pisa University, Pisa, Italy. The characteristics of the soil were the following: calcaric fluvisoil (coarse–silty, mixed, thermic, Typic Xerofluvents, pH 7.8, 18 g kg—a organic matter); Sand 32%; Silt 51%; Clay 17%; Wilting point 12.4 g/100 g; Field capacity 26.3 g/100 g; Available water 13.9 g/100 g. On May 7, 2018 on the selected trial area fertilization with 200 kg ha–1 N (ammonium sulfate 21N–0P–0K) was carried out. Turfgrass cores were secured in a polyvinyl chloride (pvc) cylinders having coarse screen bottoms and placed in the greenhouse. Cylinders were arranged in a completely randomized design with six replicates, with 7 levels of water stress (total of 42 cylinders).

The cylinders were watered by constant sub-irrigation with a water sub-irrigation level of about 3 cm, and leaves were clipped manually with scissors every 7 days to 1.5 cm. From May 15 to May 30, 2018 six experimental pvc cylinders were removed from water every three days, in order to create seven levels of water stress (16, 13, 10, 7, 4, 1, and 0 days without watering). For the last six turfgrass cylinders removed, which serve as a control (i.e., 0 days without watering), sub-irrigation was suspended the same day of the turfgrass spectral reflectance measurements, on May 31, 2018.

### Data collection

On May 31, 2018 spectral reflectance measurements of the turfgrass in each cylinder were collected indoor to prevent the multiple scattering due to canopy architecture (distortion of the biochemical signal), with the hand-held FieldSpec 4 New Generation high-resolution spectroradiometer (Analytical Spectral Devices Inc., Boulder, Co, USA), operated by the Department of Agriculture, Food, and Environment and the Department of Information Engineering, University of Pisa, Pisa, Italy. The spectroradiometer collects spectra in the 350–2,500 nm range with sampling interval of 2 nm. The nominal spectral resolution is 3 nm in the visible range and 6 nm in both the near and the short-wave infrared ranges.

The fiber optic with 25° of field of view was pointed at nadir towards the turfgrass so that the entire field of view was filled by the turfgrass itself and measurements were performed with a 50 W halogen lamp as artificial light source. Data collection using an indoor artificial light source enabled acquisition of a useful reflection signal even in those spectral ranges where major atmospheric water absorption occurs (i.e., around 1,450 nm and 1,950 nm). A standard white calibration board was employed as white reference for radiance-to-reflectance conversion and was measured every other six turfgrass measurements.

On the same day, after collection of the spectroradiometric measurements, the estimation of the following parameters was carried out:

- Volumetric Soil Water Content (SWC) (%) was measured using a Time Domain Reflectometry (TDR 350, FieldScout, soil moisture meter, Spectrum Technologies, Inc., Plainfield, USA). To determine SWC by TDR, two 12-cm-long stainless-steel rods were inserted vertically in each turfgrass core.
- Leaf Relative Water Content (LRWC) (%) was calculated on the clippings using the formula: 100 × ((FW − DW)/(TW − DW)], where FW is leaf fresh weight, TW is leaf turgid weight, and DW is leaf dry weight after oven-drying leaf samples for 72 h at 100 °C. Turgid weight was determined as weight of fully turgid leaves after soaking leaves in distilled water in the refrigerator for 24 h.
- Soil moisture (SM) (%): soil samples were collected for each core of turfgrass, weighted, put in a stove at 105–110 °C and dried to constant weight. Soil moisture (%) was calculated as follow: ((FW − DW)/FW) × 100].

### Spectral indices evaluation

The spectral reflectance collected over each turfgrass sample was processed by evaluating the spectral indices reported in Table 1, which have been shown to be the most studied and effective indices for the estimation of vegetation water content^28–31,46^. The selected indices basically involve ratios of reflectance values evaluated at two different wavelengths or normalized differences between reflectance values evaluated at two different wavelengths.

**Table 1:** Reflectance-based vegetation indices used in this study.

Vegetation index	Equation	Sensitivity	Reference
Normalized difference vegetation index (NDVI)	NDVI = (R_800_ − R_680_)/(R_800_ + R_680_)	Chlorophyll	^46^
Water index (WI)	WI = R_900_/R_970_	Relative water content	^28^
Ratio WI normalized difference vegetation index (WI/NDVI)	WI/NDVI = (R_900_/R_970_)/((R_800_ − R_680_)/(R_800_ + R_680_)]	Vegetation water content	^29^
Normalized difference water index at 1,240 nm (NDWI_1240_)	NDWI_1240_ = (R_860_ − R_1240_)/(R_860_ + R_1240_)	Vegetation water content	^30^
Normalized difference water index at 2,130 nm (NDWI_2130_)	NDWI_2130_ = (R_860_ − R_2130_)/(R_860_ + R_2130_)	Vegetation water content	^31^

### Statistical analysis

The relationship among the indices selected for the evaluation of water content and Leaf Relative Water Content (LRWC), Soil Water Content (SWC) and Soil Moisture (SM) were studied using CoStat software (CoHort, Monterey, CA, USA) and Pearson’s correlation coefficients (r) were calculated.

In particular, the correlations between LRWC and the indices interesting for detecting water stress were studied in order to verify whether some index could be useful to detect water stress in bermudagrass and, if any, to identify which are the best water stress indicators. Regression equations were studied for all the vegetation indices.

### Potential of hyperspectral multivariate analysis

An example of multivariate analysis applied to the spectral reflectance was performed to investigate the potential, within this framework, of methods accounting for the whole shape of the spectra rather than restricting the analysis to a limited number of wavelengths. Given the low number of measurements (6 for each water stress condition), more refined supervised methods such as multivariate regression or multivariate classification could not be employed. Rather, unsupervised multivariate methods were employed.

**Figure 1:**
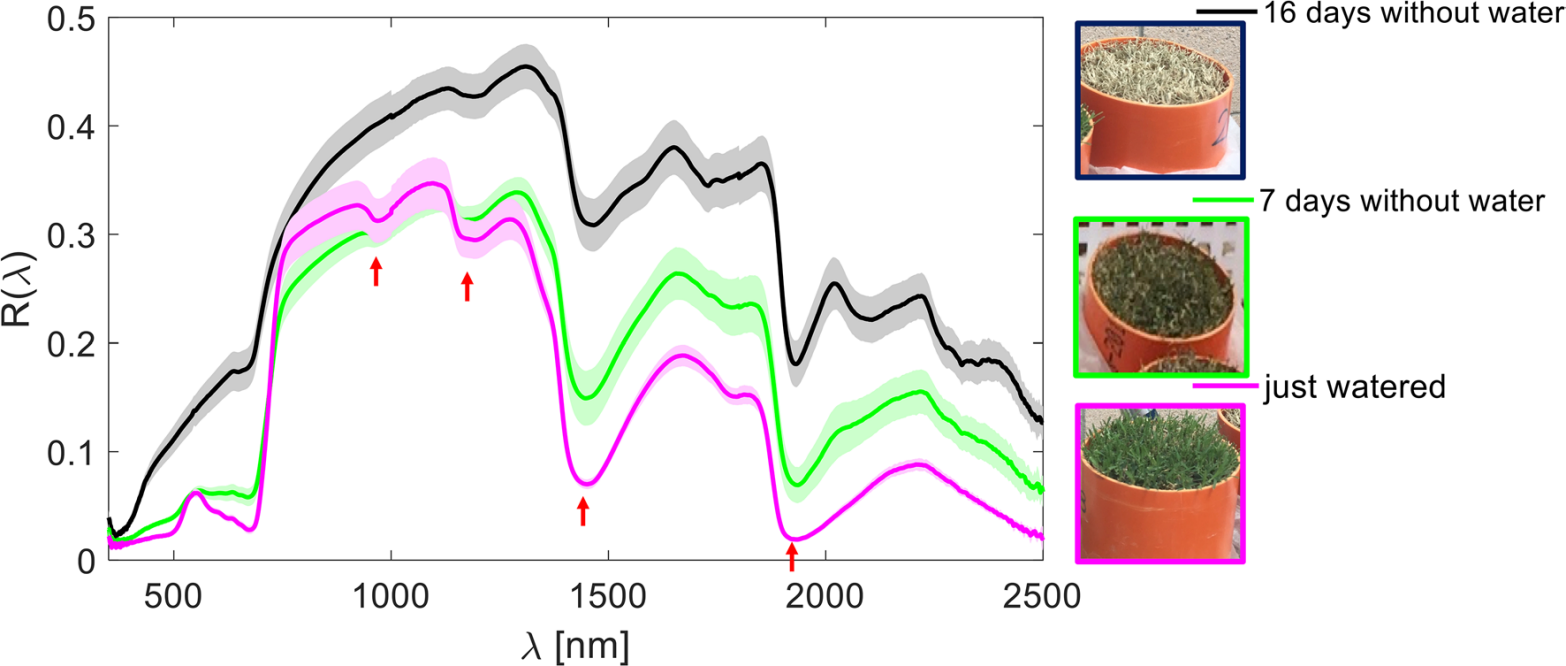
Spectral reflectance curves of bermudagrass under different levels of water deprivation: 16, 7, 0 days without watering. Each curve is the spectral reflectance averaged over the six replicates, whereas the shading shows the confidence bounds of one standard deviation around the mean. The arrows highlight the major water absorption troughs.

A PCA^43^ was first carried out. PCA performs a linear transformation to the data so that the variables in new coordinate system are uncorrelated. Specifically, the mean-centered reflectance spectra collected into the *b* × *n* matrix ***R***_0_, where *b* is the spectral dimension (#wavelengths) and *n* is the number of spectra, can be expressed as ${\varvec{R}}_{0} = {\varvec{LS}} + {\varvec{\varepsilon}}^{{\left( {\varvec{k}} \right)}}$, with ***L*** the *b* × *k* matrix of *loadings* (the PCs), ***S*** the *k* × *n* matrix of scores (the data projected into the new coordinate system), and ***ε***^(*k*)^ the residual error due to having retained *k* PCs. There may be retained up to $k = min\left( {n - 1,b} \right)$ PCs and, when *k* < *b*, the PCA acts as a dimensionality reduction technique. The PCs are orthogonal one to each other and are sorted according to a descending order of each PC contribution to the total data variance, i.e. they account for decreasing percentages of data variability. The specific PCA implementation used was the Singular Value Decomposition (SVD)^43^. Visual inspection of the loadings enabled assessment of the contribution of each wavelength to each of the PCs, whereas three-dimensional scatterplot of the scores over the first three PCs provided a visual representation of the data point cloud in the new coordinate system. In order to further investigate the potential of multivariate analysis for water stress discrimination in turfgrass, we retained three out of seven sets of turfgrass, namely those removed from sub-irrigation with a weekly frequency (i.e. those at 16, 7, and 0 days without watering, whose spectra and a representative photo are shown in Fig. 1) and performed the *k-means* spectral clustering method^43^ in the PCA-reduced three-dimensional space. The *k-means* algorithm aims at separating the data in *N* different *clusters* (subset of data with similar characteristics) without using any a priori information and trying to provide compact and separate clusters. The aim was discriminating, in a totally unsupervised fashion, among the three different water stress conditions. The spectral clustering outcome was then combined with the information brought by the spectral indices in order to label each cluster according to increasing water stress (e.g. ‘absent’, ‘medium’, ‘strong’).

## Results and discussion

**Figure 2:**
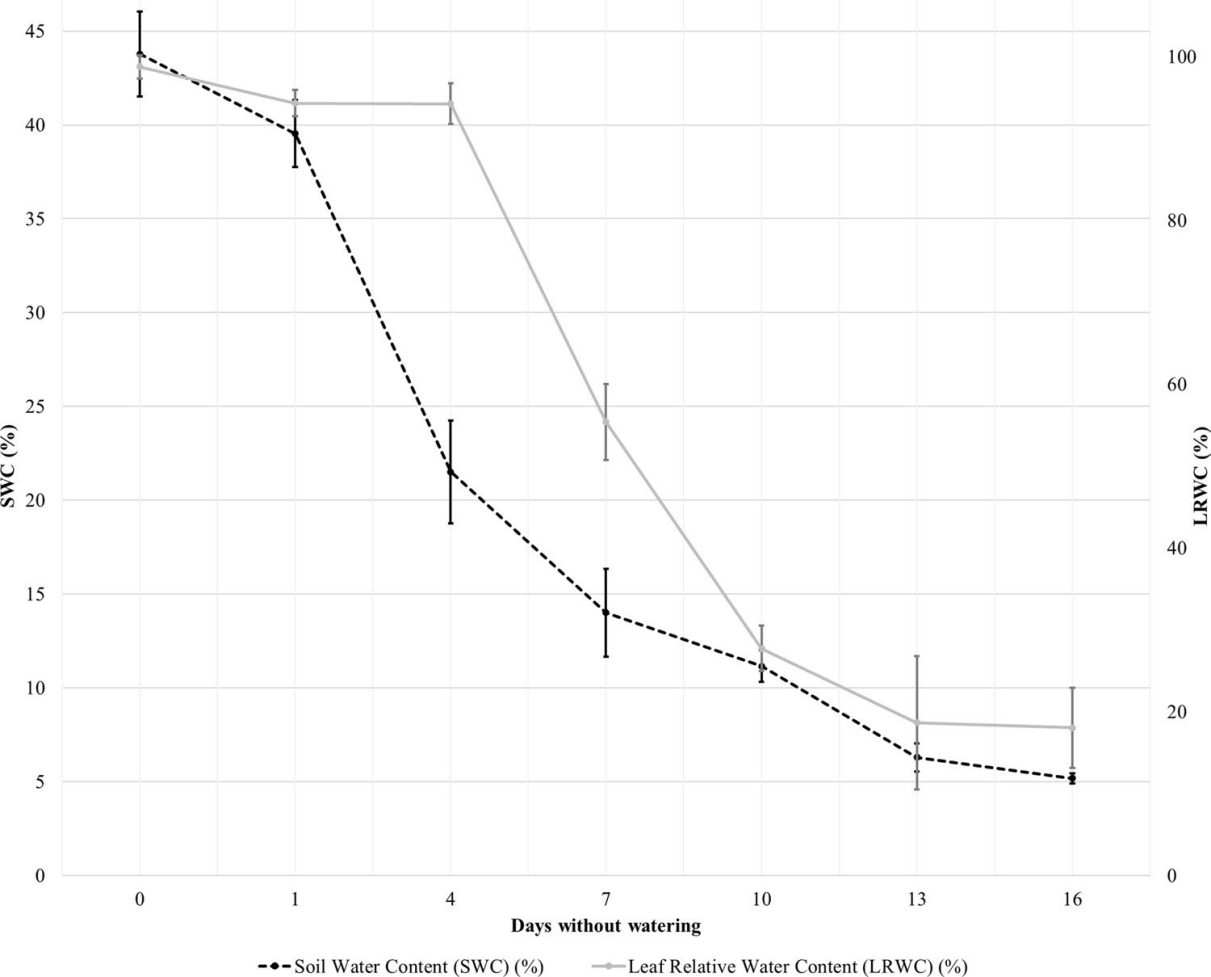
Decline in volumetric soil water content (SWC) (%) and leaf relative water content (LRWC) (%) after watering ceased. Each point is the mean of six replications. Bars indicate one standard deviation error.

Figure 1 shows the reflectance spectra collected over turfgrass at three different levels of water stress, specifically turfgrass at 16 days without watering, the intermediate situation at 7 days and at the end of the trial with the saturated cores (0 days without water), which serves as control. The differences across the curves are well evident. The major difference is the increase of reflectance at all wavelengths at 16 days without watering, where LRWC was at about 18% (Fig. 2), with respect to the other two spectral reflectance curves. It is so evident from the three different curves that in the Near-infrared (NIR 750–1,300 nm) and Short-wavelength infrared (SWIR 1,300–2,500 nm) four major absorption troughs are present. These strong reflectance troughs, located approximately in the NIR at 970 and 1,175, in the SWIR at 1,450 and 1,950 nm, are due to the absorption by water^11^. The troughs around 1,450 and 1,950 nm are less accentuated in the turf with high degree of desiccation (16 days without watering). Also González-Fernández et al.^47^ recommend calculating the band area for 1,450 nm and for 1,950 nm because of its link to equivalent water thickness, thus to estimate vine water status. Rallo et al.^48^ observed typical spectral responses in the SWIR region, where at leaf scale, absorbance bands near 1,450 and 1,900 nm could be related to the leaf water content of an olive grove.

However, in the regions of 1,350–1,480, 1,800–2,000 and 2,350–2,500 nm measurements of spectral reflectance of crop leaves are not possible in nature, also with fully sun-light conditions, because of the strong atmospheric absorption of light due to water vapor^14,32,49^ and are generally not exploited for landscape level studies. Consequently, to correctly measure these regions of wavelengths, a portable spectroradiometer system with an artificial light source must be chosen^49^. In fact, in our experiment an artificial light source was used, thus 1,430 and 1,950 can be considered key wavelengths for the measurements under artificial light source.

In the NIR spectral region there is a more commonly exploited troughs around 970 nm and in the region of 1,150–1,260, which are the most studied spectral ranges for estimation of vegetation water content^14^. It was interesting to note that the troughs of reflectance spectra underwent a gradual reduction in depth as the turfgrass desiccation increased, up to almost disappear in most cases, as showed in the 16 days without water curve. Some of the wavelengths associated with these troughs are, in fact, exploited by the spectral indices used in this study (see Table 1).

Figure 2 shows SWC and LRWC values, averaged over each set of six replicates with one standard deviation error bars, plotted with respect to the number of days without watering. Volumetric SWC declined as the days without watering increased. Starting from a value of 43.78% for the control cores with 0 days without watering, it decreased reaching a much lower value of 5.19% after two weeks without watering. Similarly, also LRWC declined as the number of days without watering increased. LRWC rate of decline was smaller than SWC as the days without watering were 4 or less (LRWC equal to 98.7%, 94.3% and 94.2% for 0, 1 and 4 days without watering, respectively). Then LRWC steeply decreased as the number of days without watering increased above 4. Observing the two parameters it is interesting to note that, with the exception of data collected in cores at 4 days without water, the trend of SWC and LRWC is similar (Fig. 2). In fact, from 1 to 4 days without water, turfgrass leaves try to preserve more water even if the soil water content decreases.

**Figure 3:**
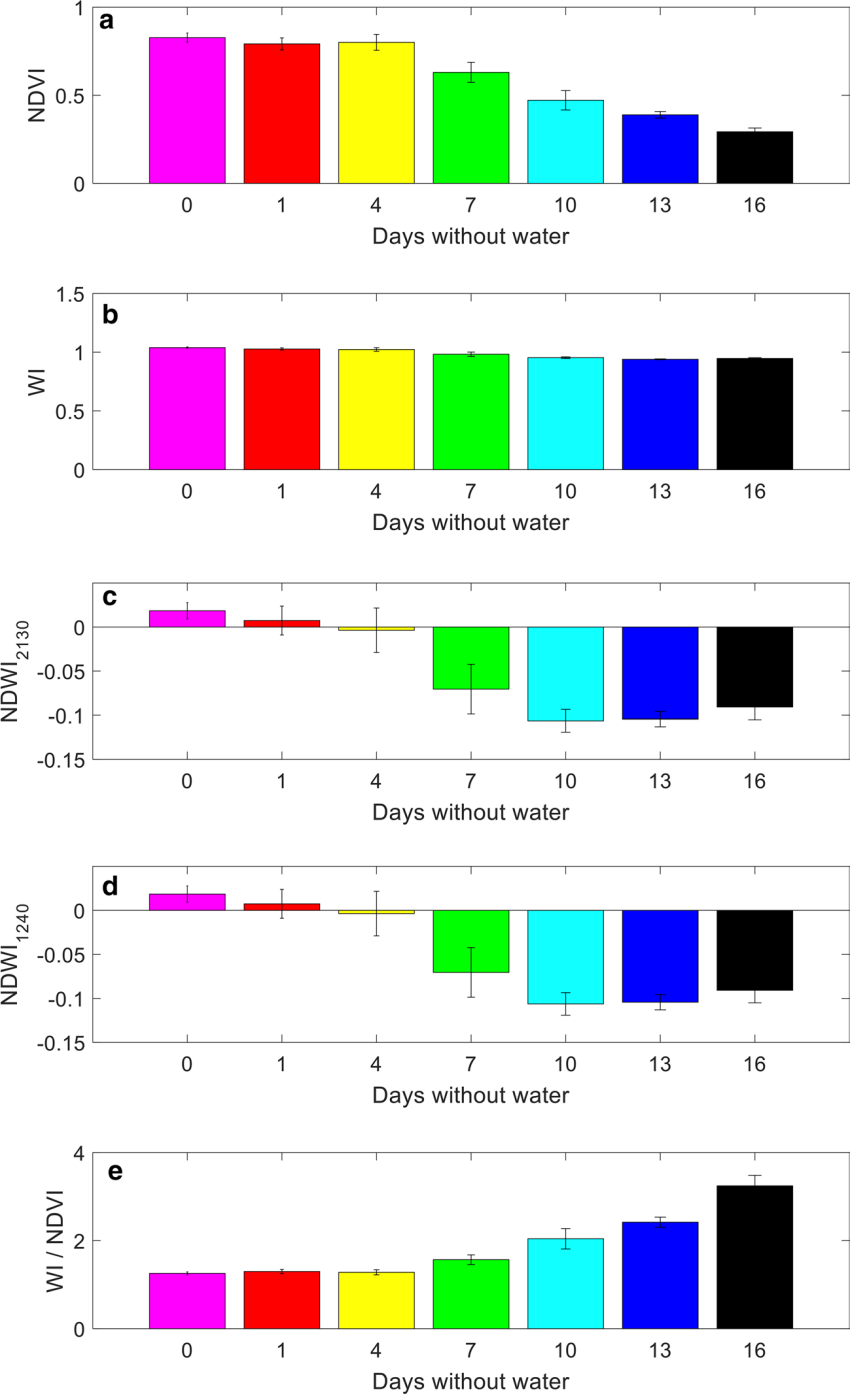
Bar graphs of spectral indices averaged over each set of six replicates at same water stress condition, with one standard deviation error bar. (**a**) NDVI, (**b**) WI, (**c**) NDWI_2130_, (**d**) NDWI_1240_, (**e**) WI/NDVI.

Figure 3 plots bar graphs of the selected indices in Table 1, where the indices are averaged over each set of six replicates of turfgrass at same water stress condition. One standard deviation error bars are also plotted. As is evident, all selected indices correlate with water stress level (Fig. 3).

A quantitative analysis of these correlations, and specifically with respect to SWC, LRWC and SM, is reported in Table 2, which reports the Pearson product-moment correlation coefficients evaluated among the various parameters and indexes studied in this work.

**Table 2:** Pearson product-moment correlation coefficients (r) among volumetric soil water content (%) (SWC) measured using a time domain reflectometry (TDR); leaf relative water content (%) (LRWC); soil moisture (%) (SM) and vegetation indices selected for the study.

r	SWC (%)	LRWC (%)	SM (%)	NDVI	WI (900/970)	WI/NDVI	NDWI_1240_	NDWI_2130_
SWC (%)	–	0.88***	0.98***	0.86***	0.89***	^A^ 0.94***	0.87***	0.88***
LRWC (%)	–	–	0.85***	0.96***	0.98***	^A^ 0.95***	0.94***	0.95***
SM (%)	–	–	–	0.82***	0.87***	^A^ 0.92***	0.87***	0.87***

Correlation coefficients are calculated across all entries.

***Significant at 0.001 level.

^A^r derived from an exponential regressive model.

### Volumetric soil water content (SWC)

**Figure 4:**
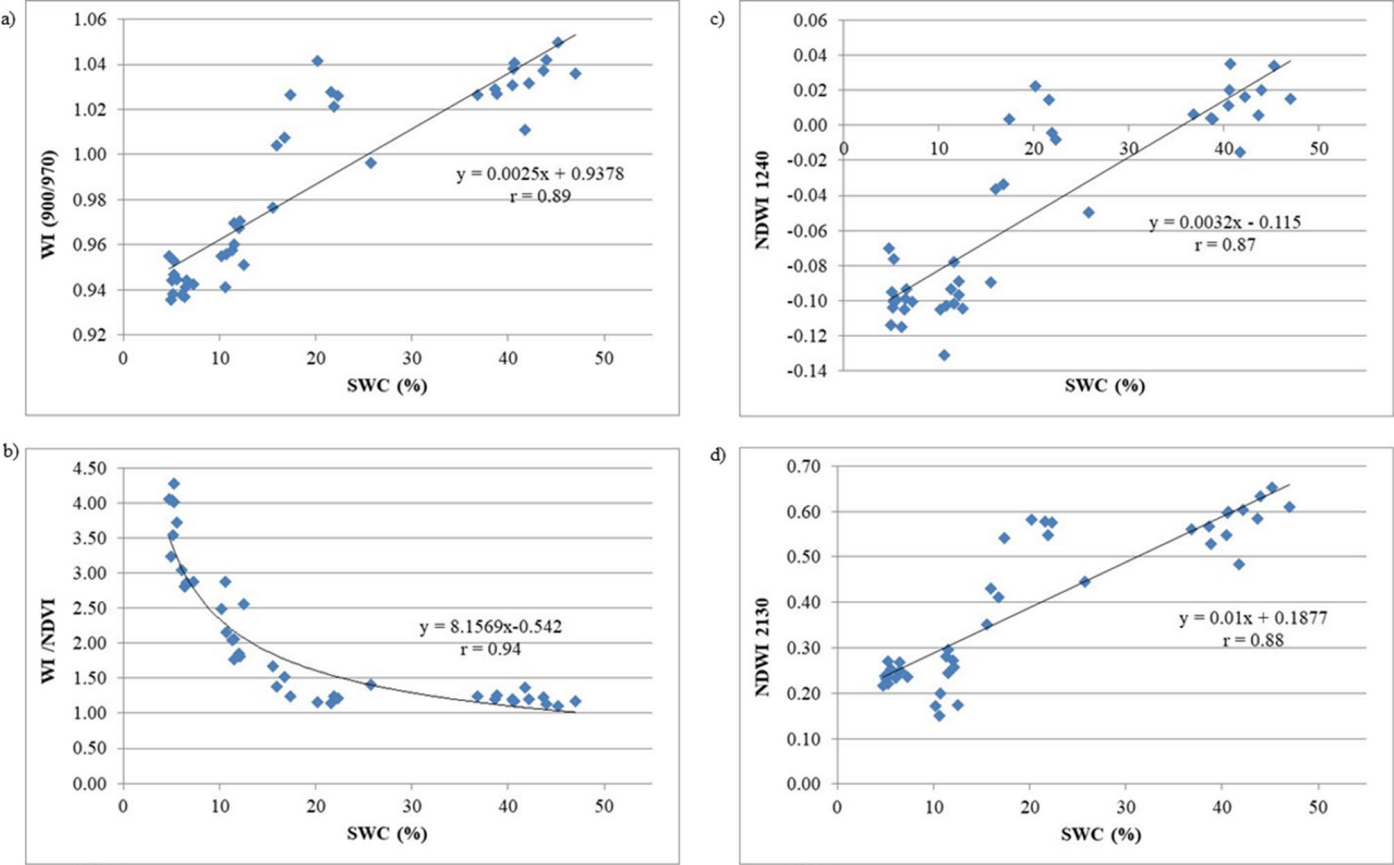
Relationship between volumetric soil water content (SWC) and (**a**) water index (R_900_/R_970_); (**b**) ratio WI normalized difference vegetation index (WI/NDVI); (**c**) normalized difference water index (NDWI_1240_); (**d**) normalized difference water index (NDWI_2130_) in Bermudagrass cores. Values represented the 6 replications.

As expected, SWC was found to be highly correlated with SM (r = 0.98, *p* < 0.001), and among the calculated indices the strongest relationship was with WI/NDVI studied by Peñuelas et al.^29^ (r = 0.94). For this relationship the exponential function proved to be the most suitable mathematical representation of the correlation. Thus, this index presented an exponential decrease when SWC values progressively increased (Fig. 4b). Relating SWC with WI, as also demonstrated by McCall et al.^3^ the correlation coefficient is still high (r = 0.89, *p* < 0.001) (Table 2; Fig. 4a). As the SWC increased to more stressed levels, also the WI and SM increased (Table 2). The range of values is between 4.75% and 47.05% of SWC corresponding respectively to a minimum WI value of 0.94 to a maximum of 1.05 (Fig. 3a). The relationships with NDWI_1240_ (r = 0.87) and NDWI_2130_ (r = 0.88) also show high coefficients (Fig. 4c,d).

### Leaf relative water content (LRWC)

For correlations (r) among LRWC and the indices selected for the evaluation of water content, significantly high r values were found for NDVI (r = 0.96), WI (900/970) (r = 0.98, *p* < 0.001), WI/NDVI (r = 0.95, *p* < 0.001), NDWI_1240_ (r = 0.94, *p* < 0.001) and NDWI_2130_ (r = 0.95, *p* < 0.001) (Table 2).

As also studied by Jiang et al.^26^ and Johnsen et al.^22^, NDVI presents a significant correlation coefficient with LRWC (r = 0.96), indicating that factors beyond water availability can impact in turfgrass quality.

As demonstrated by Peñuelas and Inoue^32^, when evaluating reflectance indices associated with water and pigment contents of peanut and wheat leaves, WI closely track changes in LRWC, but it is frequently influenced by architectural canopy parameters. Similar results were obtained by Steidle Neto et al.^50^, when assessing water and chlorophyll contents from spectral indices in sunflower plants under drought conditions. WI is effective to represent changes also in sunflower water content.

**Figure 5:**
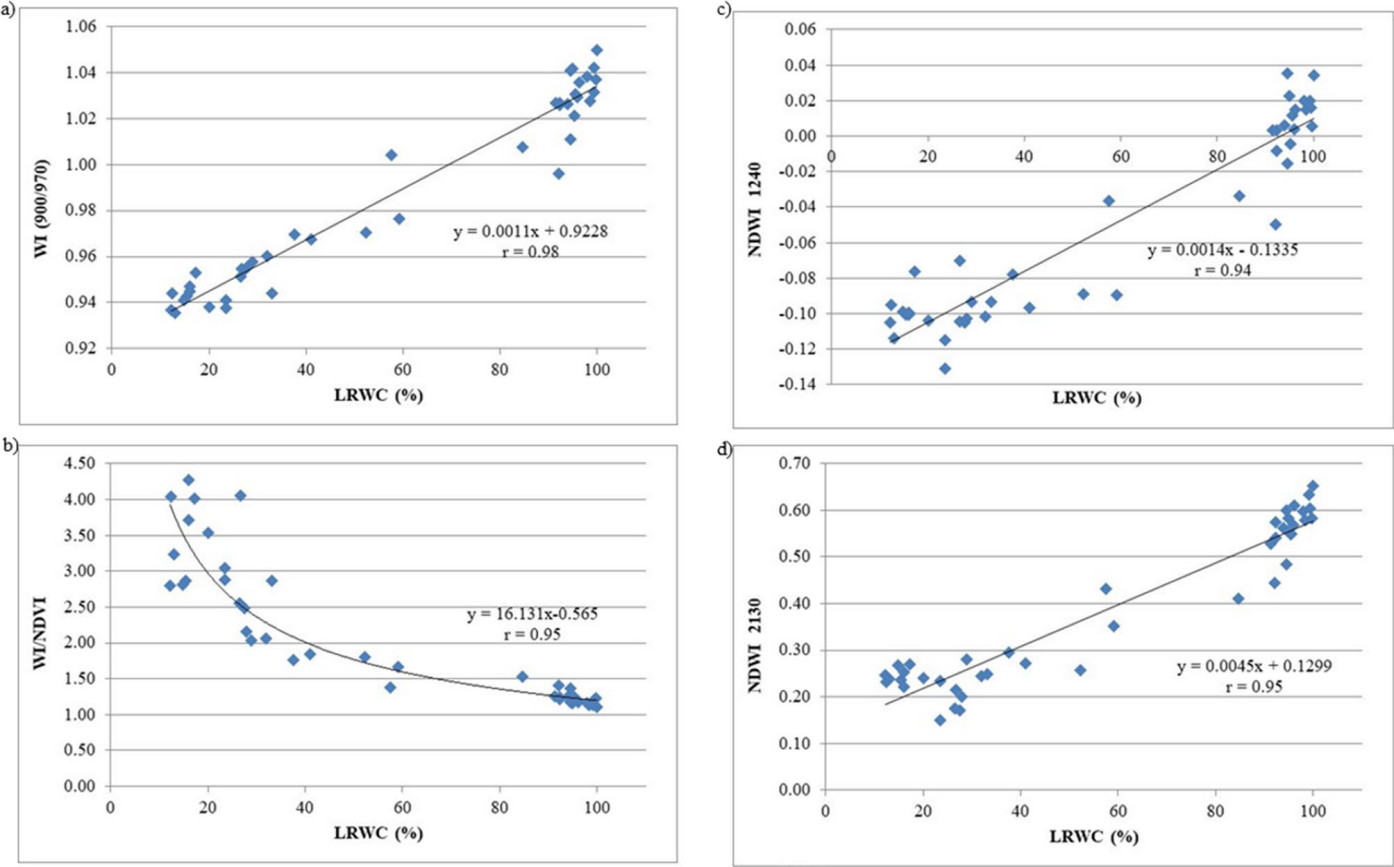
Relationship between leaf relative water content (LRWC) and (**a**) water index (R_900_/R_970_); (**b**) ratio WI normalized difference vegetation index (WI/NDVI); (**c**) normalized difference water index (NDWI_1240_); (**d**) normalized difference water index (NDWI_2130_) in Bermudagrass cores. Values represented the 6 replications.

To minimize these effects, thus maximizing the effect of vegetation water content, Peñuelas and Inoue^32^ also studied the ratio of WI with NDVI, as NDVI is an index that follows color changes in the drying leaves. Moreover, also NDWI_1240_ has registered high r value (r = 0.94, *p* < 0.001) (Fig. 4c), Gao^30^ and Serrano et al.^37^ demonstrated that NDWI together with WI showed high sensitivity to changes in canopy LRWC, better than those formulated using SWIR bands. In our research, also NDWI calculated using R_2130_, as suggested by Chen et al.^31^, showed a high correlation coefficient with LRWC (r = 0.95, *p* < 0.001) (Fig. 4d). In fact, in the SWIR, the region of 2,130–2,200 nm is one of the most suitable for measuring optical remote sensing of vegetation water content, together with the NIR wavelengths of 900, 970, and 1,150–1,260 nm band, as we can notice also in the high correlation coefficient between LRWC and NDWI_1240_ (r = 0.94, *p* < 0.001) (Table 2). Regression equations between LRWC (%) and (a) water index (R_900_/R_970_); (b) ratio WI normalized difference vegetation index (WI/NDVI); (c) normalized difference water index (NDWI_1240_) and Normalized difference water index (NDWI_2130_) in Bermudagrass cores are reported in Fig. 5.

Regarding the relationship between LRWC and WI (R_900_/R_970_) the determination coefficient was r = 0.98. Relative water content increases linearly with increasing WI with valued ranging from 0.94 to 1.05, corresponding to LRWC values ranging from 12.18% to 100% (Fig. 5a). For the relationship between LRWC and WI/NDVI the exponential function proved to be the most suitable mathematical representation of the correlation (r = 0.95). Thus, this index presented an exponential decrease when LRWC values progressively increase (Fig. 5b). In Fig. 5c, d linear regressions between LRWC and NDWI centered at different wavelengths are reported, where in both cases NDWI values increases linearly with increasing relative water content (NDWI_1240_ r = 0.94; NDWI_2130_ r = 0.95). Thus, although Serrano et al.^37^ showed that NIR-based NDWI was more sensitive to changes in canopy LRWC than those SWIR-based, in the present research the results showed that in the relationships studied between LRWC and the two NDWI, in the NIR and in the SWIR regions, the determination coefficients are significantly similar (Fig. 5c, d). Thus, they can both detect turfgrass relative water content.

### Soil moisture (SM)

**Figure 6:**
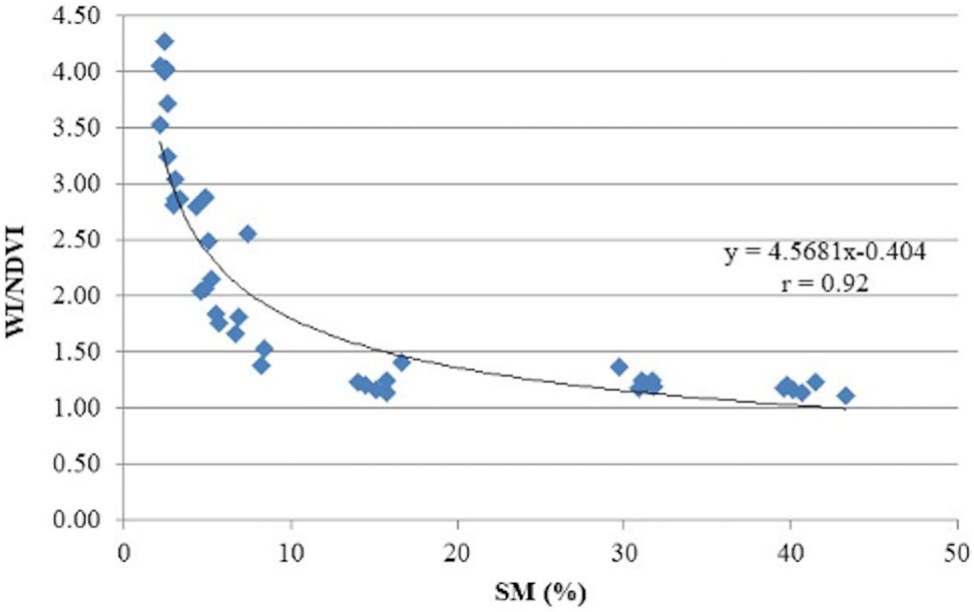
Relationship between soil moisture (SM) and ratio WI normalized difference vegetation index (WI/NDVI) in Bermudagrass cores. Values represented the 6 replications.

Regarding the relationship between soil moisture and the vegetation indices studied in the present research, the highest correlation coefficient was found with WI/NDVI (r = 0.92). In this case the exponential function proved to be the most suitable mathematical representation of the correlation. Thus, this index presented an exponential decrease when SM values progressively increase (r = 0.92) (Fig. 6).

### Potential of multivariate analysis

**Figure 7:**
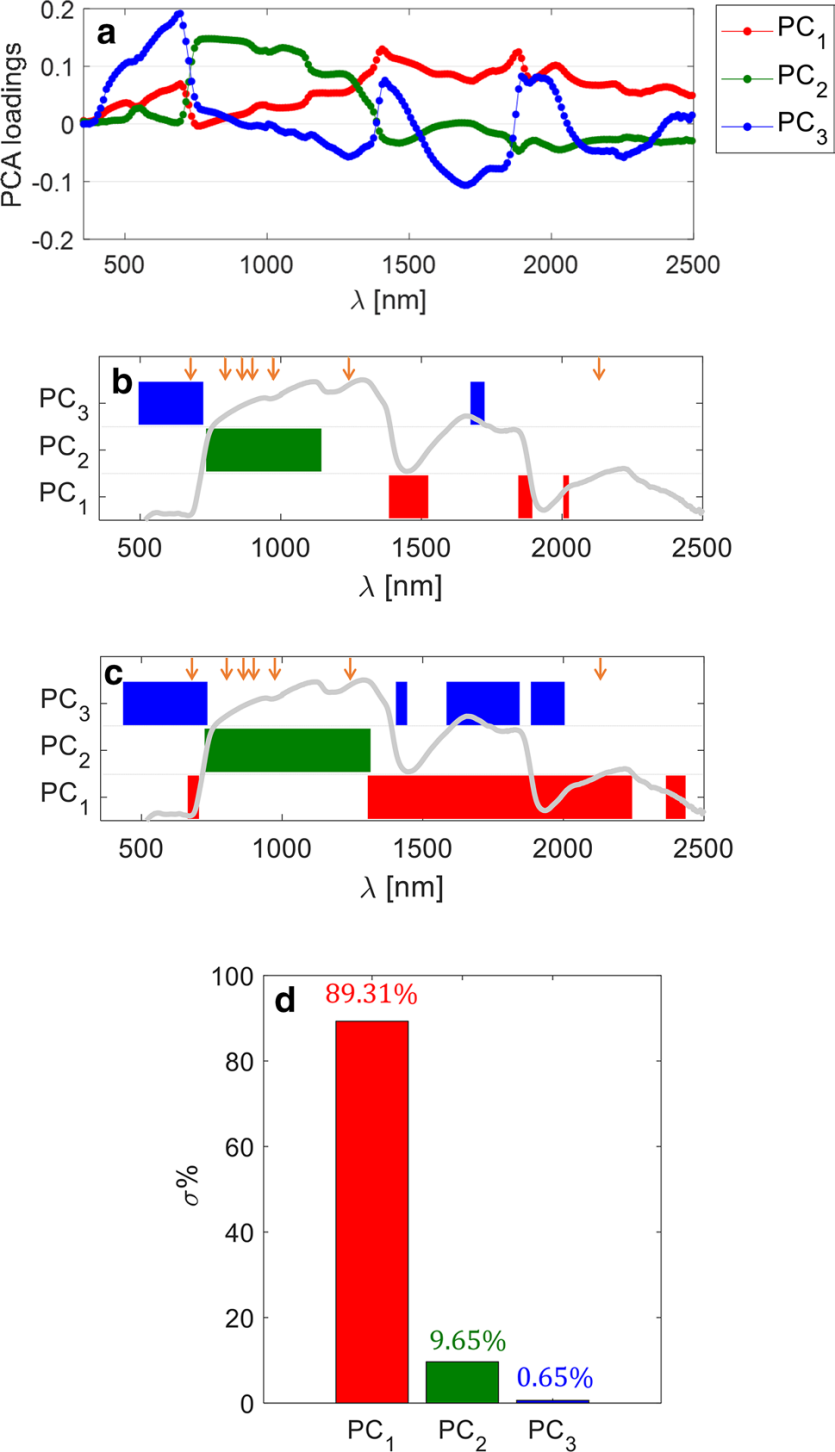
Outcomes of principal component analysis (PCA) for the first three principal components (PCs). (**a**) Spectral plots of PCA loadings. (**b**,**c**) Spectral ranges more involved in each of the first three PCs, obtained by thresholding the corresponding loadings—threshold equal to ± 0.1 for (**b**) and equal to ± 0.06 for (**c**). An example of turfgrass reflectance curve is superimposed in gray color to make result interpretation easier. The orange arrows indicate the wavelengths employed by the spectral indices used in this study. (**d**) Bar graph of the cumulative explained variance percentage.

Figure 7 shows the outcomes of the PCA, specifically as regards the first three PCs. Figure 7a displays spectral plots of the PCA loadings for each of the first three PCs (shown in different colors), plotted with respect to the wavelengths. The closer the loading at a given wavelength is to ± 1, the stronger is that specific wavelength contribution to the given PC. In order to have a better visualization of the impact of each wavelength over the PCs, Fig. 7b, c display the spectral ranges that contributed most to each of the three PCs, plotted as horizontal bars and obtained by thresholding (with two different thresholds) the corresponding loadings. To make the interpretation easier, an example of turfgrass spectral reflectance (and, specifically, the average reflectance for the 7 days without watering condition) is plotted on the same graphs. The figures clearly show that the ranges contributing most to the first PC (in red) are most of the short-wave infrared, with the water absorption bands around 1,450 and 1900 nm providing the strongest contributions. The near infrared band is the range contributing most to the second PC (in green), where the visible range and part of the short-wave infrared provide the strongest contributions to the third PC (in blue). As is evident, the wavelengths used by the spectral indices employed in this study and highlighted with orange arrows in Fig. 7b, c, are included within these spectral ranges. However, there are several other wavelengths that, according to PCA, are strongly “informative” and are worth being exploited for water stress monitoring. For completeness, Fig. 7d plots a bar graph of the cumulative percentage of explained variance by the first three PCs. The first PC by itself explains about 89% of spectra variability and the three PCs together explain more than 99% of it.

**Figure 8:**
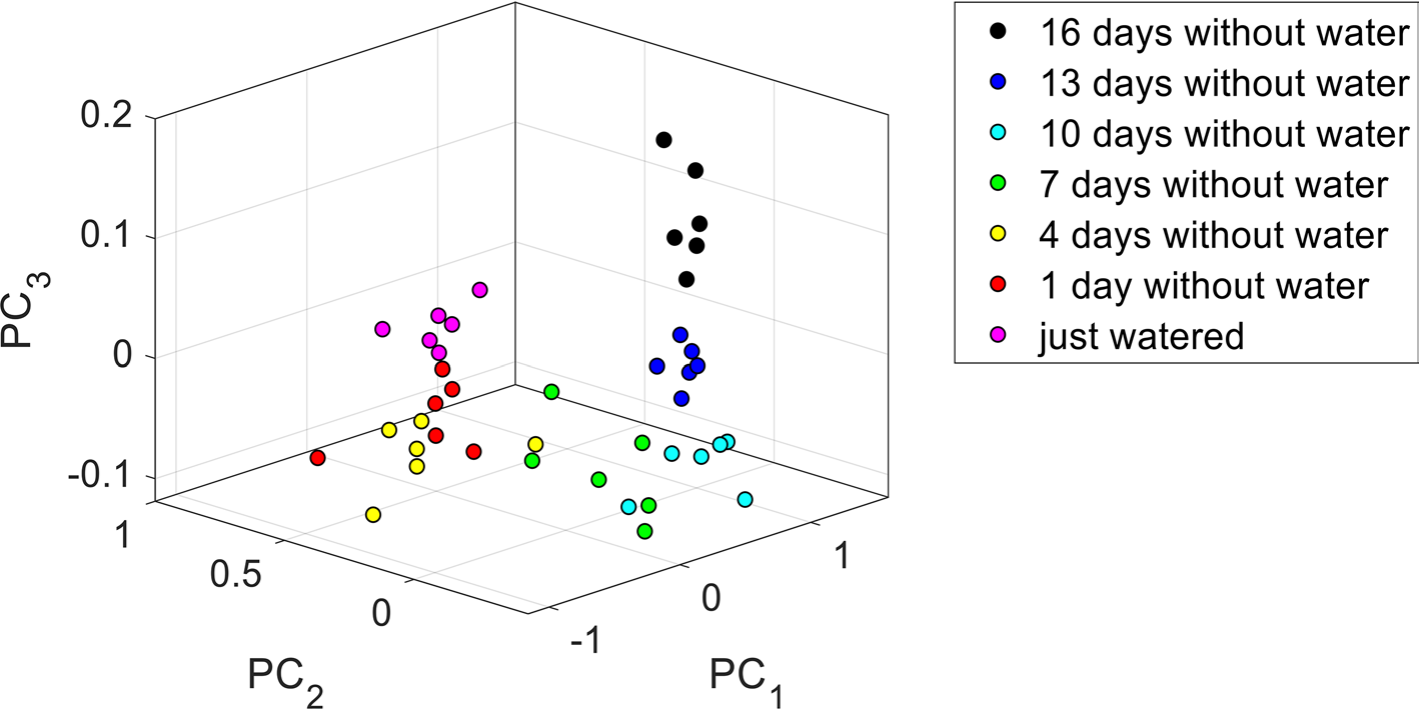
Scatterplot of the spectra of turfgrass at different water stress levels over the three first PCs.

Figure 8 plots a scatterplot of the turfgrass reflectance spectra in the three-dimensional space spanned by the first three PCs. Although the reflectance data place themselves in the space following a rather complex data structure, the data are mostly arranged in a sort of ordered fashion with respect to water stress, i.e. data related to similar stress conditions are closer to each other within the data structure, whereas data related to different stress conditions are placed apart to each other.

**Figure 9:**
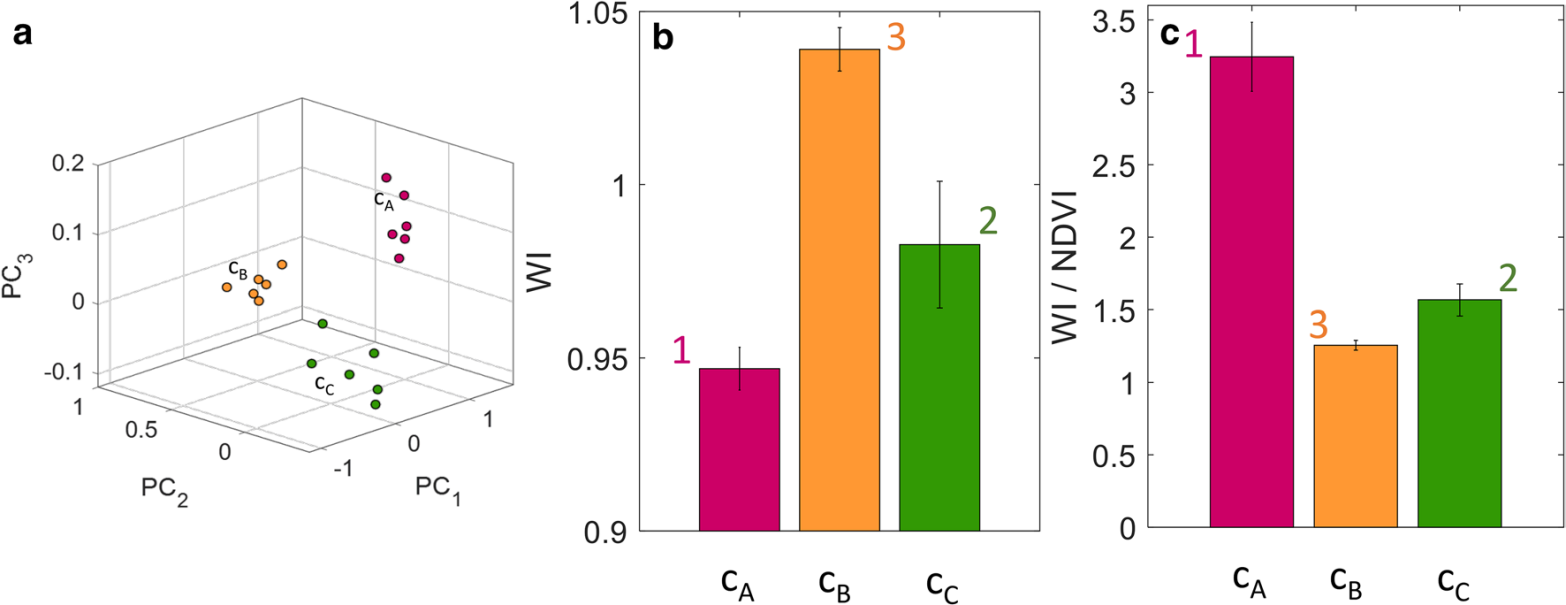
(**a**) Scatterplot of the result of the *k*-means clustering method applied with *N* = 3 clusters. The method succeeded in correctly identifying the three groups of turfgrass. (**b**) Bar graph of WI index averaged over each identified cluster (with one standard deviation error bar). An ascending order of WI indicates a descending order of water stress level. (**c**) Bar graph of WI/NDV index averaged over each identified cluster (with one standard deviation error bar). A descending order of WI/NDVI indicates a descending order of water stress level.

Application of the *k-means* multivariate clustering method, applied with *N* = 3 clusters to the data subsets related to the aforementioned ‘strong’ (16 days without water), ‘medium’ (7 days without water), and ‘absent’ (just watered ) water stress conditions resulted in a correct identification of the three different groups, as shown in Fig. 9a where a scatterplot of the three data subset is shown and the different colors denote the different clusters obtained with *k*-means. By comparing Fig. 9a with Fig. 8, it is evident that the clusters denoted in Fig. 9a with *c*_*A*_, *c*_*B*_, and *c*_*C*_, corresponds respectively to the ‘strong’, ‘absent’, and ‘medium’ stress conditions. However, although having separated correctly the three subsets of data, application of *k*-means by itself does not tell us anything about the water stress condition of each subset of data. Evaluation of the average of both WI (900/970) and WI/NDVI indices (which have revealed to be, among the indices studied here, the more effective indicators of water stress) over the three clusters identified with *k*-means allowed the clusters to be sorted according to a descending order of water stress level (i.e., according to an ascending order of WI or descending order of WI/NDVI, respectively), as shown in Fig. 9b,c.

Although performed on a data set limited in terms of sample size, this analysis has shown that multivariate methods have great potential for water stress monitoring in turfgrass and surely deserve further investigations.

## Conclusions

The sustainable use of resources and optimization of inputs, such as irrigation and fertilization, are basic also in the management and maintenance of turfgrasses. In fact, drought stress in turfgrass is one of the main abiotic stresses influencing turfgrass growth and quality.

Several studies have been carried out to provide different methods for estimating the vegetation water content at different scales: ground-based or remotely sensed measurements.

NDVI is the most commonly used vegetation index also to assess water stress but it uses narrow bands that overlap with chlorophyll features. This research demonstrates that there are different vegetation indices and in particular WI (900/970), WI/NDVI, NDWI_1240_, NDWI_2130_ that can be used as vegetation water stress indicator, in this case on bermudagrass grown in the Mediterranean area (central Italy). The relationships studied, suggest high correlations coefficients (r) among the studied indices and the selected parameters. WI (900/970) and WI/NDVI indices have revealed to be, among the indices studied here, the more effective indicators of water stress. In fact, lower values of WI indicate higher water stress both in leaves and soil, while WI/NDVI at higher values correspond higher water stress levels.

In future it could be interesting to test the same new generation spectroradiometer on different turfgrasses to verify if the selected indices are similarly effective at detecting water stress across different species.

A simple example of hyperspectral multivariate analysis has also been shown that has revealed the great potential of multivariate methods for water stress monitoring in turfgrass.

Data showed in the present research refers to a trial conducted in a greenhouse with controlled conditions and the spectroradiometric measurements were carried out indoor with an artificial light source. Future researches could be conducted with the same spectroradiometer on turfgrass directly in the field under sunlight conditions, to test the new generation instrument but also to verify if the selected indices are valid and effective also when spectra are acquired outdoor. Even though the strong atmospheric water vapor absorption is likely to impair, in outdoor conditions, the proper calculation of some of the selected indices, an outdoor campaign will also allow the collection of a much higher number of spectra, thus enabling application of more sophisticated multivariate methods, such as supervised classification methods and multivariate regression approaches.
